# Gold Nanoparticles Enhancing Generation of ROS for Cs-137 Radiotherapy

**DOI:** 10.1186/s11671-022-03761-w

**Published:** 2022-12-14

**Authors:** Shiao-Wen Tsai, Chang-Yun Lo, Shang-Yang Yu, Fang-Hsin Chen, Hsiao-Chieh Huang, Lu-Kai Wang, Jiunn-Woei Liaw

**Affiliations:** 1 Department of Biomedical Engineering, Chang Gung University, Taoyuan, Taiwan; 2 Department of Periodontics, Chang Gung Memorial Hospital, Taipei, Taiwan; 3 Department of Mechanical Engineering, Chang Gung University, Taoyuan, Taiwan; 4 Department of Medical Imaging and Radiological Sciences, Chang Gung University, Taoyuan, Taiwan; 5 Department of Radiation Oncology, Chang Gung Memorial Hospital, Taoyuan, Taiwan; 6 Institute of Nuclear Engineering and Science, National Tsing Hua University, Hsinchu, Taiwan; 7 Proton and Radiation Therapy Center, Linkou Chang Gung Memorial Hospital, Taoyuan, Taiwan; 8 Radiation Biology Core Laboratory, Institute for Radiological Research, Chang Gung University/Chang Gung Memorial Hospital, Taoyuan, Taiwan; 9 Department of Mechanical Engineering, Ming Chi University of Technology, New Taipei City, Taiwan

**Keywords:** Cs-137, Radiotherapy, Gold nanoparticles, Reactive oxygen species, Radiosensitizer, Disruption of cytoskeleton, Mitochondrial damage, Amplification factor, Radiosensitization enhancement factor, Tumoricidal efficacy

## Abstract

Radiotherapy is an important modality for the treatment of cancer, e.g., X-ray, Cs-137 *γ*-ray (peak energy: 662 keV). An important therapy pathway of radiation is to generate the double strand breaks of DNA to prohibit the proliferation of cancer cells. In addition, the excessive amount of reactive oxygen species (ROS) is induced to damage the organelles, which can cause cellular apoptosis or necrosis. Gold nanoparticles (GNPs) have been proven potential as a radiosensitizer due to the high biocompatibility, the low cytotoxicity and the high-*Z* property (*Z* = 79) of gold. The latter property may allow GNPs to induce more secondary electrons for generating ROS in cells as irradiated by high-energy photons. In this paper, the radiobiological effects on A431 cells with uptake of 55-nm GNPs were studied to investigate the GNPs-enhanced production of ROS on these cells as irradiated by Cs-137 *γ*-ray. The fluorescence-labeling image of laser scanning confocal microscopy (LSCM) shows the excessive expression of ROS in these GNPs-uptake cells after irradiation. And then, the follow-up disruption of cytoskeletons and dysfunction of mitochondria caused by the induced ROS are observed. From the curves of cell survival fraction versus the radiation dose, the radiosensitization enhancement factor of GNPs is 1.29 at a survival fraction of 30%. This demonstrates that the tumoricidal efficacy of Cs-137 radiation can be significantly raised by GNPs. Because of facilitating the production of excessive ROS to damage tumor cells, GNPs are proven to be a prospective radiosensitizer for radiotherapy, particularly for the treatment of certain radioresistant tumor cells. Through this pathway, the tumoricidal efficacy of radiotherapy can be raised.

**Supplementary Information:**

The online version contains supplementary material available at 10.1186/s11671-022-03761-w.

## Introduction

Radiotherapy utilizing high-energy photon (e.g., X-rays and Cs-137 *γ*-ray) beam or proton beam is a useful modality for cancer treatment. Two mechanisms of radiotherapy causing the apoptosis or even necrosis of tumor cells were extensively studied; the double strand break (DSB) of DNA in cells by ionization, and the damage on cellular organelles by the produced reactive oxygen species (ROS), e.g., hydroxyl free radicals [1–4]. Recently, a variety of radiosensitizers to enhance the efficacy of radiotherapy has been developed [5–7]. In particular, using gold nanoparticles (GNPs) to produce excessive ROS for raising the tumoricidal efficacy of radiation therapy has attracted a lot of attentions [8–15]. Since gold is a high *Z* material (*Z* = 79) with good biocompatibility and low cytotoxicity, GNP is a prospective candidate as radiosensitizer [12–16]. Various GNPs with different shapes, sizes and coatings (surface modifications) were developed to increase the cellular uptake and used as radiosensitizer for X-rays, Gamma ray of Cs-137 and even proton therapy [17–24]. A few of papers have shown that ultrasmall GNPs (e.g., 2–6 nm) can pass through nuclear pore into nucleus, whereas larger GNPs (size > 10 nm) only stay in cytoplasm [21]. In addition, several previous research works demonstrated that bare and spherical GNPs with an average diameter of 50 to 55 nm are more easily internalized by cells with low cytotoxicity [22, 25–27]. Recently, Chithrani et al. found that 50-nm GNPs perform the maximum radiosensitization enhancement factor (REF) of 1.66 at 10% cell survival fraction (SF) for the radiation of X-ray of 105 kVp [22]. In contrast, the REFs are 1.48, 1.18, and 1.17 for the radiations of X-ray of 220 kVp, Cs-137 *γ*-ray (662 kVp), and X-ray of 6 MVp, respectively; i.e., the higher the photon energy, the lower the REF of GNPs [22]. This is because that the K-shell binding energy of electrons in gold is 80.7 keV. Additionally, Enferadi et al. used ultrasmall GNP-PEG (size: 2.6 nm) as radiosensitizer for different radiation sources: X-rays, Gamma ray of Cs-137 *γ*-ray and proton beam. Their results show that GNP-PEG provides a significant enhancement on all these radiotherapies [20]. As we know, GNPs are internalized via endocytosis, and then certain number of GNPs are aggregated and enclosed in thousands of vehicles within cytoplasm via organelle fusion [22, 27]. A few of studies used Monte Carlo simulation to investigate the mechanism of radiosensitization of GNPs on enhancing radiobiological efficacy [28–35]. In principle, the ionizing radiation can induce ejected electrons from GNPs through Auger effect, Compton effect, and photoelectric effect [36, 37]. The ejected electrons (photoelectrons, Auger electrons) and ionized electrons in water can produce excessive free radicals, particularly ROS [36]. Consequently, the excessive ROS cause the cellular damage, resulting in apoptosis and necrosis [38–46]. The relationship between the disruption of cytoskeleton and the excessive ROS induced in GNPs-uptake cells irradiated by a femtosecond laser (two-photon effect) has been verified [27]. This is to say that the damage on organelles by excessive ROS is another pathway to kill tumor cells, except the DSB of DNA.

In this paper, we quantitatively study the REF of non-targeted GNPs of 55-nm size on Cs-137 *γ*-ray radiotherapy from the curve of cell SF versus radiation dose [20, 22, 47]. We aim to provide more biological evidences to elucidate the mechanism of GNPs-enhanced ROS inducing the damage of organelles (mitochondria and cytoskeletons) to cause apoptosis or necrosis. Our results may pave a way to using GNPs as radiosensitizer to increase the production of ROS for raising the tumoricidal efficacy of radiotherapy, which might be useful to treat certain radioresistant tumor cells.

## Method and Materials

We synthesized GNPs with an average size of 55 nm according to the previous synthesis recipe [27]. Additionally, the concentration of GNPs was measured by an inductively coupled plasma atomic emission spectroscopy (Agilent 5110) for experimental preparation. For our experiments, GNPs colloids of different concentrations were prepared. In our study, physics and biology experiments were conducted, individually. Two systems of Cs-137 *γ*-ray were used for radiation: MDS: Gammacell 40 Exactor (Canada) and Varian Medical Systems (UK). In fact, the radiation sources of Cs-137 are the same for both systems; the configuration of the Gammacell 40 Exactor facilitates the experiments of samples in tubes, and the configuration of Varian Medical Systems is for the experiments of cells on culture plates. The former was used for the experiments of inducing ROS in GNP colloid and the cell viability (clonogenic assay), and the latter was for the experiments of the damage of cellular organelles. For physics experiment, GNPs suspension (aqueous solution) was irradiated by Cs-137 (MDS: Gammacell 40 Exactor) for the measurement of ROS. An enzyme-linked immunosorbent assay (ELISA) reader (SpectraMax i3x, Molecular Devices, USA) was used to measure ROS with a labeling kit of Carboxy-H2DCFDA. In the presence of ROS, the DCFH of this kit can be converted to DCF, which is highly fluorescent as being excited by a light within the region of 488 nm to 495 nm.

**Fig. 1:**
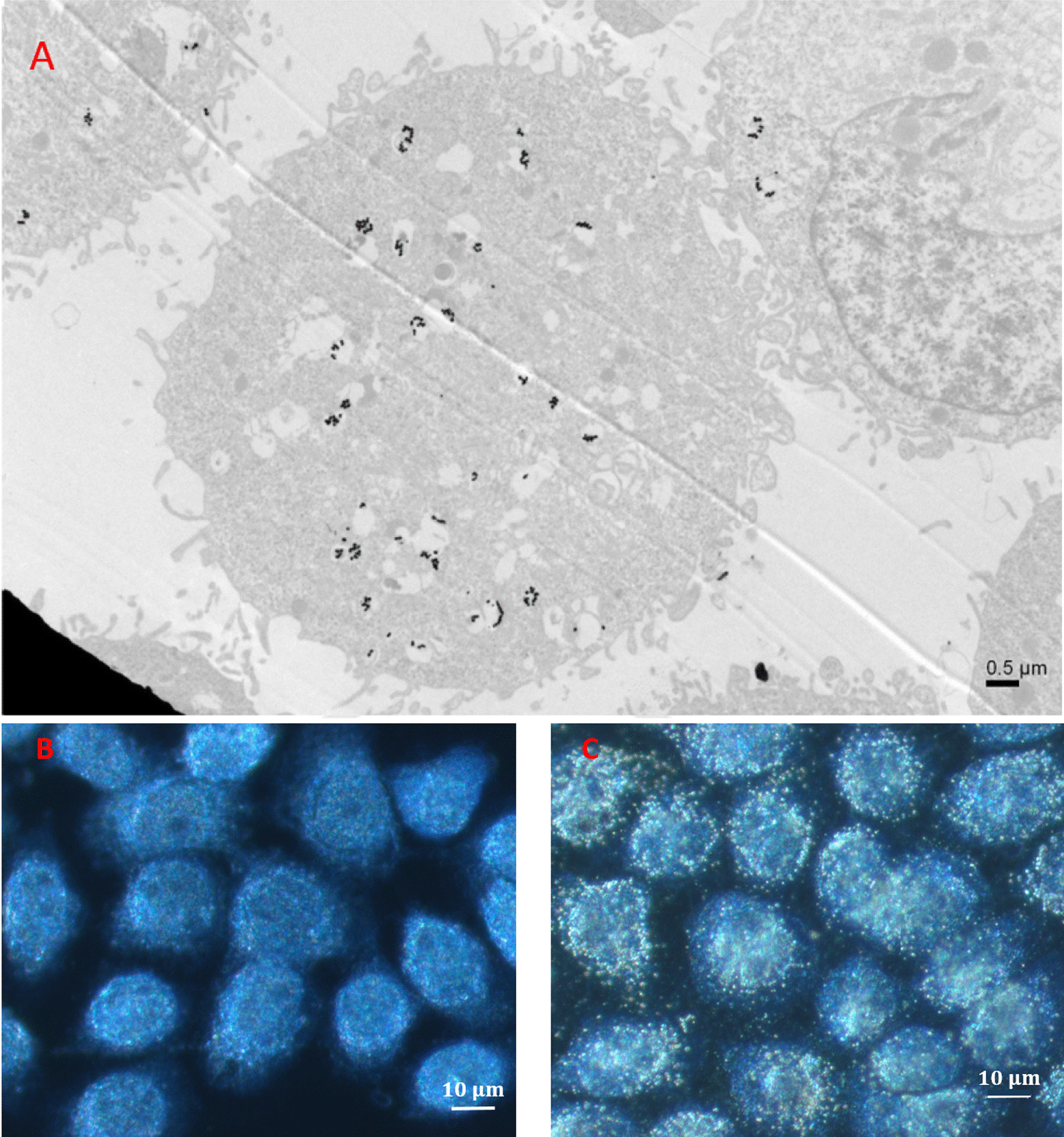
**A** TEM image of GNPs-uptake cells. Black dots in the image are GNPs; certain number of GNPs are enclosed in vesicles through the endocytosis and fusion. **B** and **C** the dark-field microscope images of the controls and GNPs-uptake cells. Bright spots in **C** are the light scattering of these GNPs contained in vesicles. In contrast, there is no light scattering in controls

For biology experiments, cell line of A431, human epidermoid carcinoma, was used for our experiment [27]. These cells were co-cultivated with a medium of 80-ppm GNPs for 24 h in advance of our experiments. Figure 1A shows the transmission electron microscope (TEM) image of cells with GNPs uptake, where the aggregation of GNPs enclosed by vesicles in cytoplasm is caused by the endocytosis and vesicle fusion [48]. The dark-field microscope (100×, ZEISS) image of cells with GNPs uptake is shown in Fig. 1C, where the bright spots in cytoplasm are due to the scattered light from these vesicles with certain number of GNPs. Figure 1B is the image of the controls without GNPs.

For the LSCM image, the nuclei of cells are stained by Hoechest 33,342, and the ROS labeling kit is Carboxy-H2DCFDA. Additionally, the kits for labeling mitochondria and cytoskeletons are MitoCapture™ and Alexa Fluor™ 488 Phalloidin, respectively. A laser scanning confocal microscopy (LSCM) (ZEISS LSM 780 META) is used to acquiring the cellular fluorescence images for detecting the labeling ROS or organelles. The excitation wavelength of laser and the emission passband of filter for exciting and detecting the fluorescence of different kits for the images of LSCM are listed in Additional file 1: Table S1. Notice that the colors in the images of LSCM are the pseudocolors not the real ones of fluorescence.

## Results and Discussion

**Fig. 2:**
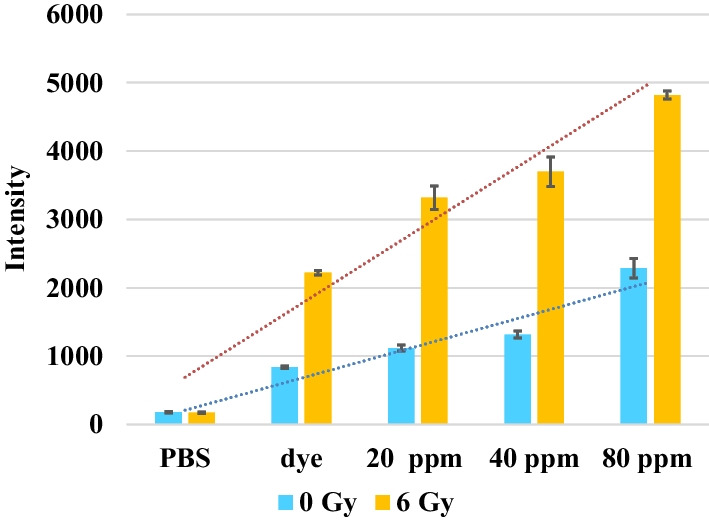
Fluorescence expression of ROS kit in GNPs suspension versus various concentrations irradiated by 6 Gy Cs-137. Kit for ROS: Carboxy-H2DCFDA. Excitation wavelength: 495 nm, and emission wavelength: 529 nm for ELISA. Dash lines: linear regression

First, the amounts of ROS produced in GNPs suspensions of different concentrations irradiated by 6 Gy Cs-137 were measured by ELISA reader, as plotted in **Fig. ****2**. The linear regressions of the intensity of ROS kit (*y*) for dose 0 Gy and 6 Gy in terms of the concentration (*x*) of GNPs are 469*x*-258.8 and 1076.8*x*-384.8, respectively. The former can be regarded as the baseline. The slope of the line of 6 Gy is significantly larger than that of the control (0 Gy). These results demonstrate that the amount of produced ROS in aqueous solution of GNPs is increased as the concentration increases.

### Biological Experiments

**Fig. 3:**
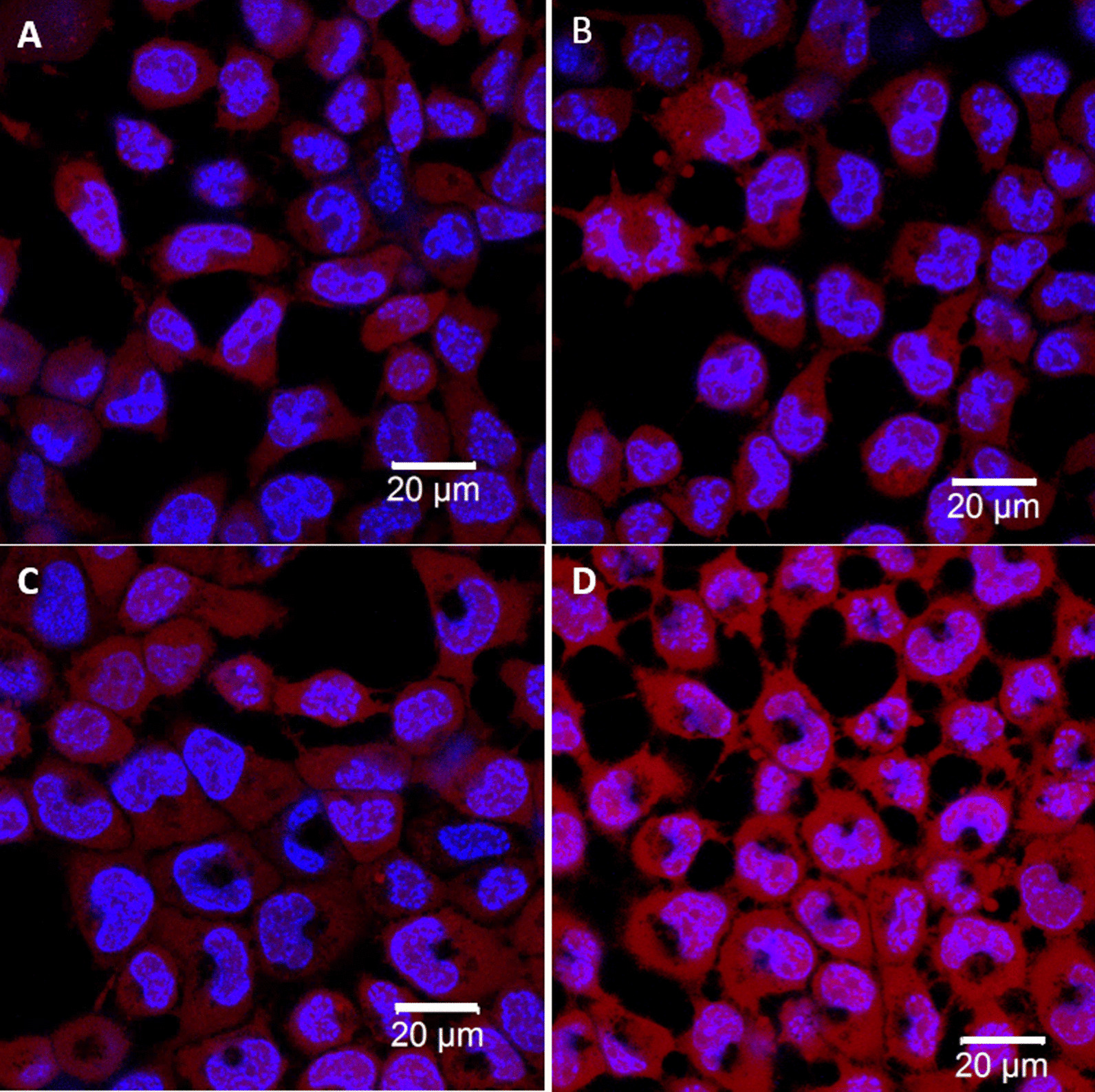
Cell fluorescence images of LSCM for ROS kit irradiated by Cs-137 with a dose of 6 Gy (magnification: × 63). **A** and **B** are the images of the controls without and with irradiation of Cs-137, respectively. **C** and **D** the images of the GNPs-uptake cells without and with irradiation, respectively. Kit for ROS: Carboxy-H2DCFDA (red). Excitation laser: 488 nm; emission filter: 509–535 nm. Kit for nuclei: Hoechest 33,342 (blue)

First, the cells of A431 were co-cultivated with a medium of 80-ppm GNPs for 24 h in advance. Subsequently, these GNPs-uptake cells were irradiated by Cs-137 with a dose of 6 Gy. Right after the irradiation, the fluorescence expression of kit (Carboxy-H2DCFDA) for labeling ROS in these cells measured by LSCM is shown in Fig. 3 (magnitude: ×63). The conditions of LSCM are listed in Additional file 1: Table S1. In addition, a low-magnitude (×20) image is shown in Additional file 1: Fig. S1. The results demonstrate that after the irradiation, the amount of ROS in these GNPs-uptake cells is significantly higher than that in the control group, as shown in Fig. 3B and D. Although the lifetime of ROS is short, the induced damages of ROS (oxidative stress) on the organelles are long term.

**Fig. 4:**
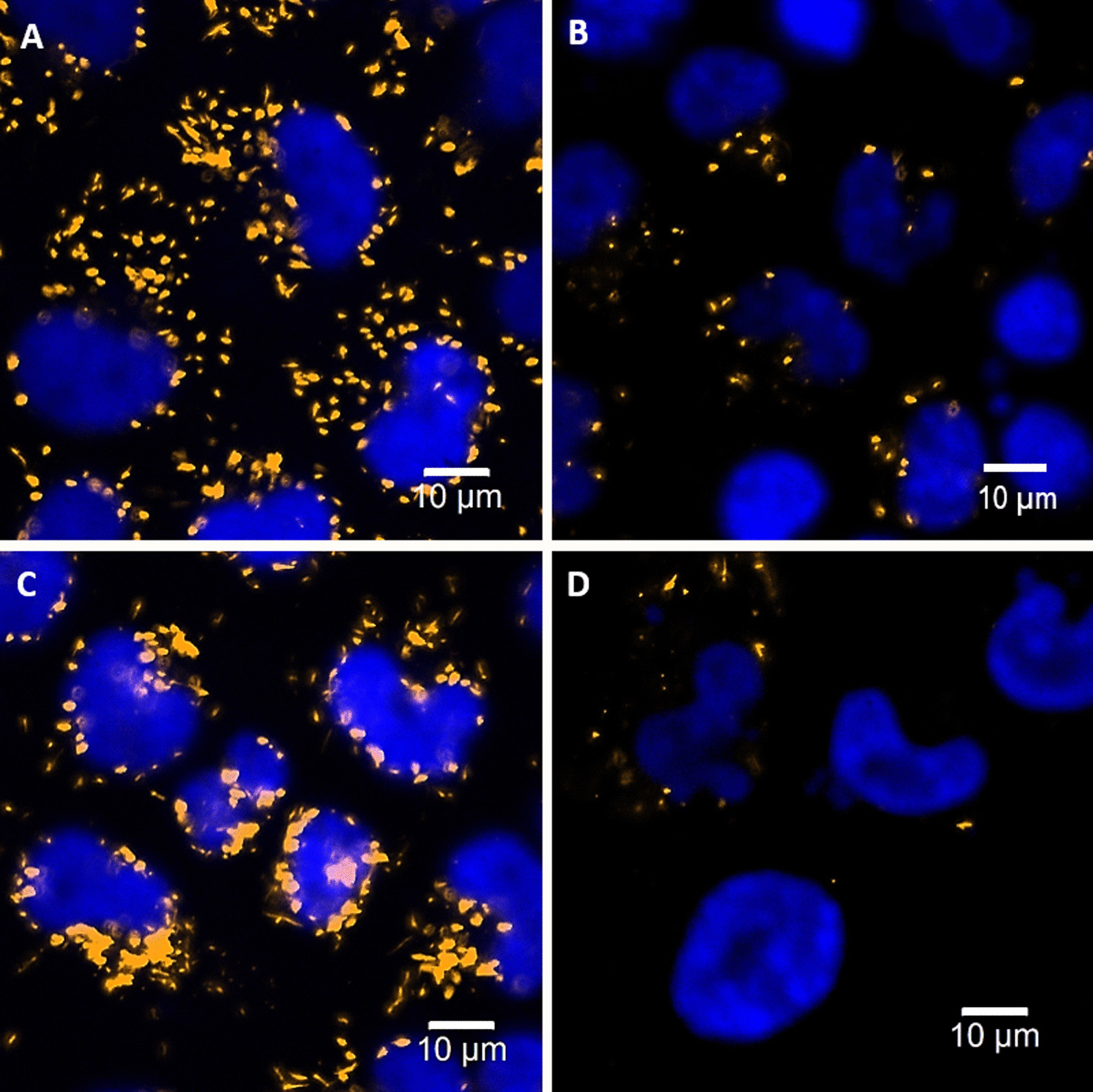
Cell fluorescence images of LSCM for labeled activate mitochondria, 48 h after irradiation of 6 Gy Cs-137 (magnification: × 100). **A** and **B** are the images of the controls without and with irradiation of Cs-137, respectively. **C** and **D** are the images of the GNPs-uptake cells without and with irradiation, respectively. Kit for mitochondria: MitoCapture™ (yellow). Kit for nuclei: Hoechest 33,342 (blue)

Furthermore, we investigated the ROS-induced damages on cellular organelles (e.g., mitochondria and cytoskeletons) by using LSCM. In Fig. 4 (magnification: ×100), the bright spots in these cell images represent the fluorescence expression of active mitochondria (labeled by MitoCapture™) in cells, 48 h after the irradiation of 6 Gy Cs-137 (Varian Medical Systems). Obviously, the numbers of active mitochondria in the controls and the GNPs-uptake cells exposed to the radiation are significantly reduced, in comparison with these cells without irradiation. Moreover, the number of active mitochondria in these GNPs-uptake cells is obviously lower than that of the controls, as irradiated by Cs-137 (Fig. 4B and D). This is an evidence of the mitochondrial damage caused by the excessive ROS generated from GNPs in these cells, as irradiated by Cs-137. In addition, a low-magnitude (×20) image is shown in Additional file 1: Fig. S2. This phenomenon is due to that the excessive ROS could cause the dysregulation of mitochondria, including the damage of mitochondrial DNA [49].

**Fig. 5:**
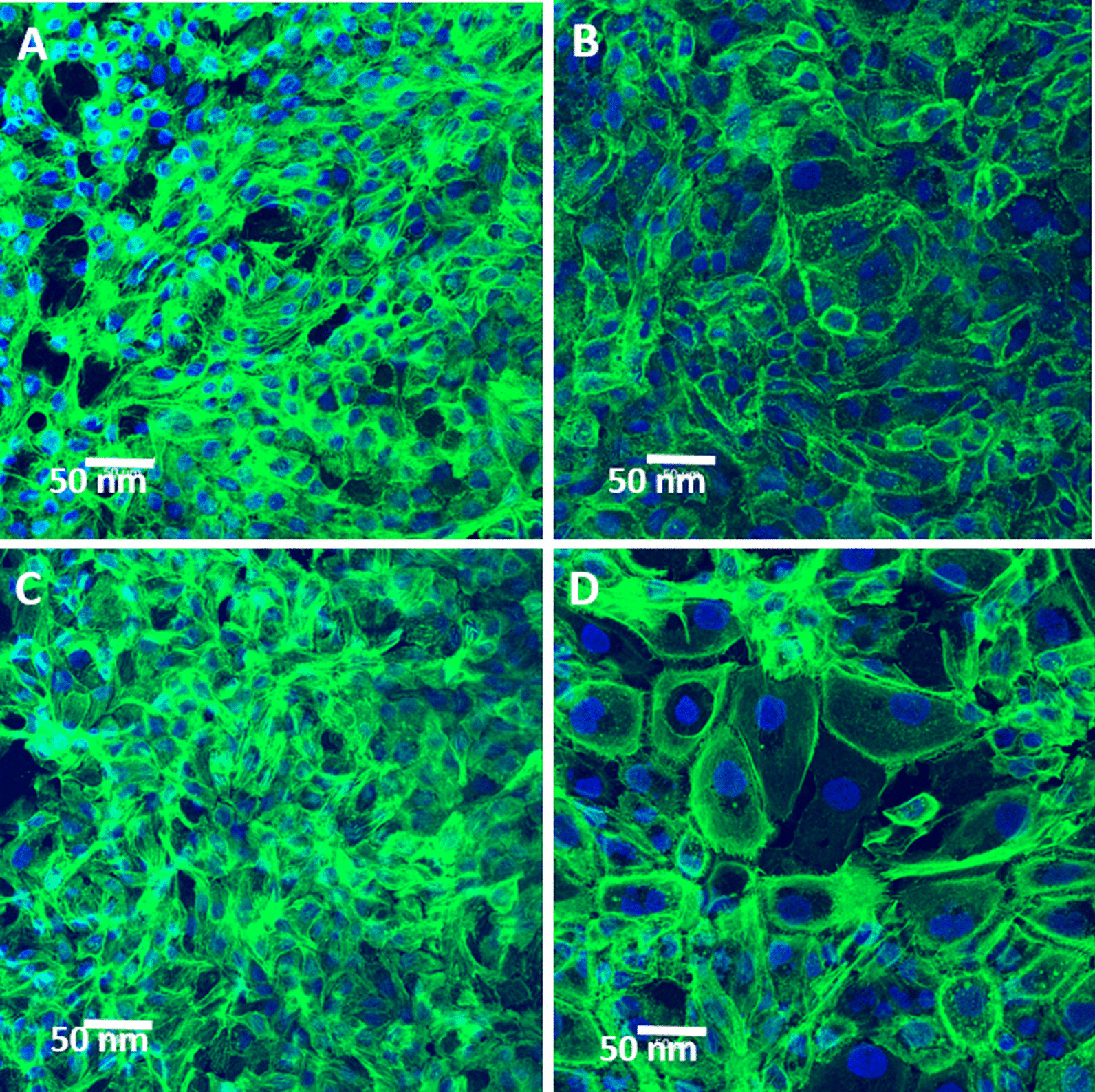
Cell fluorescence images of LSCM for labeled cytoskeletons, 48 h after the irradiation of Cs-137 with a dose of 6 Gy (magnification: × 20). **A** and **B** are the images of the controls without and with irradiation of Cs-137, respectively. **C** and **D** the images of the GNPs-uptake cells without and with irradiation, respectively. Kit for cytoskeletons: Alexa Fluor™ 488 Phalloidin (green). Kit for nuclei: Hoechest 33,342 (blue)

We also investigated the ROS-induced damage on the cytoskeletons. Figure 5 (magnification: ×20) shows the fluorescence expression (green) of the cytoskeletons in cells, labeled by Alexa Fluor™ 488 Phalloidin. Before irradiation, the integrities of the cytoskeletons of the controls and the GNP-uptake cells are almost the same. However, the disruptions of cytoskeleton in these GNPs-uptake cells are more severe than those in the controls, 48 h after the irradiation of 6 Gy Cs-137 (Varian Medical Systems), in comparison with the control. This could be a consequence of cytoskeleton disruption caused by the excessive ROS, e.g., hydroxyl free radicals. The reason is that ROS can induce the depolymerization of actin filaments. As we know, cytoskeleton consists of actin filaments, intermediate filaments, and microtubules. The major function of cytoskeleton is to maintain the cell shape. Hence, once the integrity of cytoskeleton is broken by the excessive ROS, the cellular swelling is induced. A high-magnitude (×100) image is shown in Additional file 1: Fig. S3. Additionally, the morphology of a bigger nucleus could be due to the incomplete mitosis, which is an early indication of apoptosis caused by DNA damage.

**Fig. 6:**
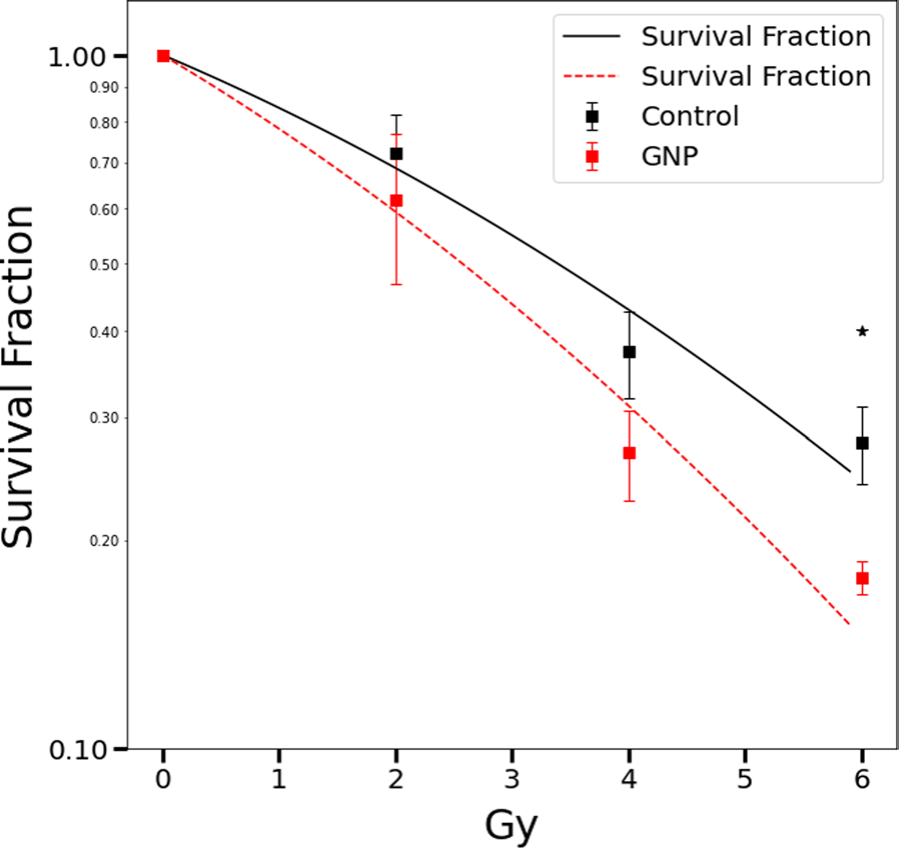
Cell viability (in vitro clonogenic assay) of A431 with GNPs uptake; cell SF (8 days after Cs-137 irradiation) versus radiation dose. Control group: cells without GNPs co-culture. ★: P < 0.02

Furthermore, we used the clonogenic assay method to measure the viability of A431 cells in vitro, 8 days after Cs-137 (MDS: Gammacell 40 Exactor) irradiation of different doses (0, 2, 4, 6 Gy). Using a fitting model of exponential decay in terms of a function of a linear and quadratic forms of dose, we obtain the estimated curves of the SFs of the GNPs-uptake cells and the control. Figure 6 shows the curves of SFs for the GNPs-uptake cells and the control (without GNPs co-cultivation) versus radiation dose. From the two curves, the amplification factor (AF) of GNP on cell SF at a specific dose is defined as the ratio of SF difference,

1 $${\text{AF}} = \frac{{{\text{SF}}_{{{\text{control}}}} - {\text{SF}}_{{{\text{with}}\;{\text{ GNPs}}}} }}{{{\text{SF}}_{{{\text{control}}}} }} \times 100\%$$

The AFs at 2, 4 and 6 Gy are 13.6%, 28.2% and 36.1%, respectively. In addition, REF of GNPs on Cs-137 is defined as the ratio of the dose without GNPs to the dose with GNPs at a specific SF of 30%; REF is 1.292 [20, 22]. Not only AFs but also REF quantitatively illustrate that 55-nm GNPs are potential radiosensitizers for enhancing radiation therapy of Cs-137, as listed in Table 1.

In summary, our results show that ROS expressions in the 55-nm GNPs suspensions and in the GNPs-uptake cells irradiated by Cs-137 are significantly higher than those in the controls. Furthermore, the cell images of LSCM indicate the corresponding disruption of cytoskeletons in these GNPs-uptake cells is more severe, compared to the control. In addition, the number of active mitochondria in these cells is dramatically reduced. The REF of GNPs on Cs-137 therapy at a SF of 30% is 1.29. The qualitative biological evidence and the quantitative REF prove the feasibility of using GNPs as radiosensitizer for Cs-137 radiotherapy.

## Conclusion

In this paper, the efficacy of GNPs to increase the production of ROS under the irradiation of Cs-137 and the radiobiological effects on cells were studied. The cell images of LSCM verify the excessive expression of ROS produced in these cells with GNP uptake as being irradiated by Cs-137 radiation. Consequently, the significant disruption or damage of cytoskeletons and mitochondria, caused by the excessive ROS, in these cells were also observed by LSCM. Except the directional damage on DNA, the excessive ROS could cause the indirect damage on cellular organelles, e.g., mitochondria and cytoskeletons, to induce the apoptosis. According to the curves of cell SF versus radiation dose of Cs-137, the REF of GNPs is 1.29, which exhibits a significant enhancement on the tumoricidal efficacy of Cs-137 therapy. Our radiobiological results may pave a way to using GNPs as radiosensitizer to increase the production of ROS for raising tumoricidal efficacy of radiotherapy. This GNP-assisted radiotherapy might be particularly useful to treat certain radioresistant tumor cells. The In the future, the technique of surface modification, e.g., PEG, folic acid or peptide, on GNPs may be used to enhance the cellular uptake of GNPs for multi-functional medical applications of image, drug delivery, radiotherapy and so on [20, 22, 50–52].

**Table 1:** AFs and REF of GNPs on Cs-137 therapy calculated from cell SF versus radiation dose

	AF (2 Gy)	AF (4 Gy)	AF (6 Gy)	REF at 30%
Cs-137 662 keV	13.60%	28.20%	36.13%	1.292

## Supplementary Information

**Additional file 1**. **Table S1**: Parameters setup of the wavelengths of the excitation (Ex) lasers and the passbands of the emission (Em) filters of LSCM for inducing and detecting the fluorescence of different biomarkers (kits) for ROS and organelles in cells. **Fig. S1**: Cell fluorescence images of LSCM for labeled ROS irradiated by Cs-137 with a dose of 6 Gy (magnification: ×20); **Fig. S2**: Cell fluorescence images of LSCM for labeled activate mitochondria, 48 hours after irradiation of 6 Gy Cs-137 (magnification: ×20); Fig. S3: Cell fluorescence images of LSCM for labeled cytoskeletons, 48 hours after the irradiation of Cs-137 with a dose of 6 Gy (magnification: ×100).

## Data Availability

All the data and material are available in the manuscript.

## Abbreviations

GNPs: Gold nanoparticles
ROS: Reactive oxygen species
LSCM: Laser scanning confocal microscopy
DSB: Double strand breaks
SF: Survival fraction
REF: Radiosensitization enhancement factor
AF: Amplification factor
ELISA: Enzyme-linked immunosorbent assay
TEM: Transmission electron microscope
