# Supplementary material for: Gold Nanoparticles Enhancing Generation of ROS for Cs-137 Radiotherapy
Source: Nanoscale Res Lett. 2022 Dec 14;17:123. doi: 10.1186/s11671-022-03761-w (PMC9751242; doi:10.1186/s11671-022-03761-w)
Supplement: Supplementary file 1 — Additional file 1. Table S1: Parameters setup of the wavelengths of the excitation (Ex) lasers and the passbands of the emission (Em) filters of LSCM for inducing and detecting the fluorescence of different biomarkers (kits) for ROS and organelles in cells. Fig. S1: Cell fluorescence images of LSCM for labeled ROS irradiated by Cs-137 with a dose of 6 Gy (magnification: ×20); Fig. S2: Cell fluorescence images of LSCM for labeled activate mitochondria, 48 hours after irradiation of 6 Gy Cs-137 (magnification: ×20); Fig. S3: Cell fluorescence images of LSCM for labeled cytoskeletons, 48 hours after the irradiation of Cs-137 with a dose of 6 Gy (magnification: ×100). [file 11671_2022_3761_MOESM1_ESM.docx]

**Supplementary Information**

**Gold Nanoparticles Enhancing Generation of ROS for Cs-137 Radiotherapy**

Shiao-Wen Tsai^1,2^, Chang-Yun Lo^3^, Shang-Yang Yu^3^, Fang-Hsin Chen^4,5,6^, Hsiao-Chieh Huang^5,7^, Lu-Kai Wang^8^, Jiunn-Woei Liaw^3,7,9^*

^1^Department of Biomedical Engineering, Chang Gung University, Taoyuan, Taiwan

^2^Department of Periodontics, Chang Gung Memorial Hospital, Taipei, Taiwan

^3^Department of Mechanical Engineering, Chang Gung University, Taoyuan, Taiwan

^4^Department of Medical Imaging and Radiological Sciences, Chang Gung University, Taoyuan, Taiwan

^5^Department of Radiation Oncology, Chang Gung Memorial Hospital, Taoyuan, Taiwan

^6^Institute of Nuclear Engineering and Science, National Tsing Hua University, Taiwan

^7^Proton and Radiation Therapy Center, Linkou Chang Gung Memorial Hospital, Taoyuan, Taiwan

^8^Radiation Biology Core Laboratory, Institute for Radiological Research, Chang Gung University/Chang Gung Memorial Hospital, Taoyuan, Taiwan

^9^Department of Mechanical Engineering, Ming Chi University of Technology, New Taipei City, Taiwan

Corresponding author: [markliaw@mail.cgu.edu.tw](mailto:markliaw@mail.cgu.edu.tw)

Various kits for labeling reactive oxygen species (ROS) and organelles for the images of laser scanning confocal microscopy (LSCM) are listed in Table S1. The parameters setup for cellular fluorescence-label images of LSCM is listed in Table S1, including the excitation wavelengths of lasers for inducing different fluorescence of biomarkers (kits) and the passbands of the filters for detecting the emission of the corresponding fluorescence. The ROS in water includes singlet oxygen, superoxide, hydroxyl radical and so on. The kit for labeling ROS is Carboxy-H2DCFDA. In the presence of ROS, the DCFH of the kit is converted to DCF, which is highly fluorescent as being excited by a laser of 488 nm. The colors of different fluorescence of in these images are pseudo colors, rather than the true colors. The cell fluorescence images of LSCM for labeled ROS irradiated by Cs-137 with a dose of 6 Gy (magnification: ×20) are shown in Fig. S1. Fig. S2 shows the cell fluorescence images of LSCM for labeled activate mitochondria, 48 hours after irradiation of 6 Gy Cs-137 (magnification: ×20). Fig. S3 shows the cell fluorescence images of LSCM for labeled cytoskeletons, 48 hours after the irradiation of Cs-137 with a dose of 6 Gy (magnification: ×100).

**Table S1** Parameters setup of the wavelengths of the excitation (Ex) lasers and the passbands of the emission (Em) filters of LSCM for inducing and detecting the fluorescence of different biomarkers (kits) for ROS and organelles in cells.

|  | ROS | Cytoskeletons | Mitochondria | Nuclei |
| --- | --- | --- | --- | --- |
| Biomarkers (kits) | Carboxy-H2DCFDA | Alexa Fluor^TM^ 488 Phalloidin (25λ+1000λ PBS) | MitoCapture (2λ in buffer 1 ml) | Hoechst 33342 (4λ in PBS 1 ml) |
| LSCM  Ex/Em  (nm) | 488/509-535 | 488/500-553 | 488/580-598 | 405/410-472 |


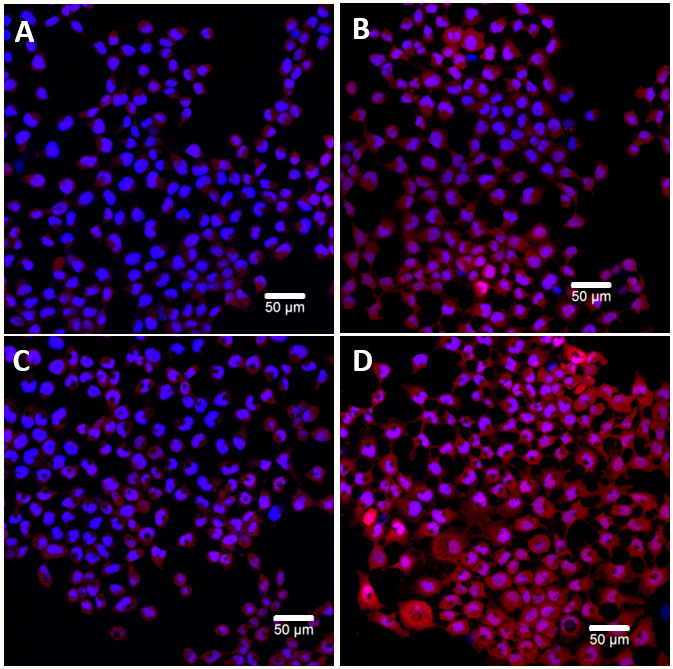


**Fig. S1** Cell fluorescence images of LSCM for labeled ROS irradiated by Cs-137 with a dose of 6 Gy (magnification: ×20). **A** and **B** are the images of the controls without and with irradiation of Cs-137, respectively. **C** and **D** the images of the GNPs-uptake cells without and with irradiation, respectively. Kit for ROS: Carboxy-H2DCFDA (red). Kit for nuclei: Hoechest 33342 (blue).


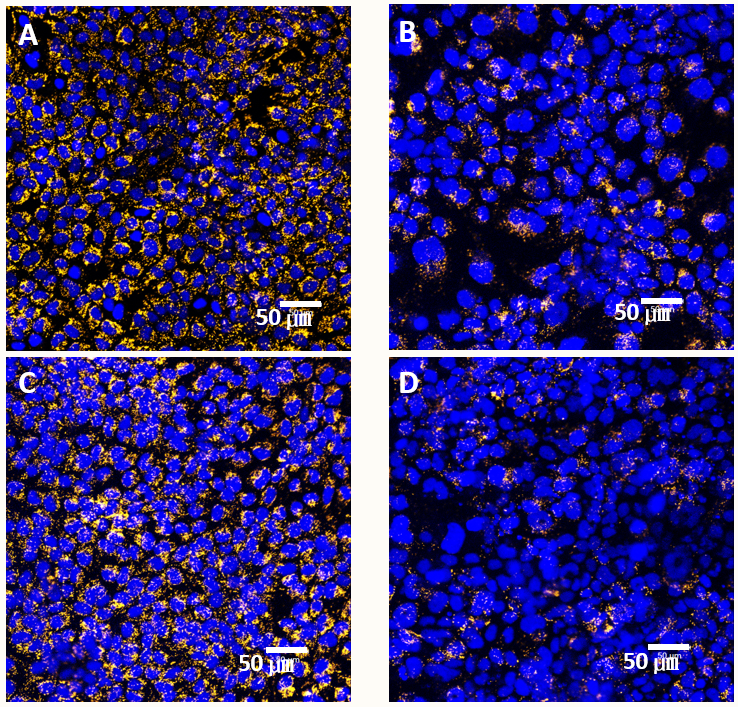


**Fig. S2** Cell fluorescence images of LSCM for labeled activate mitochondria, 48 hours after irradiation of 6 Gy Cs-137 (magnification: ×20). **A** and **B** are the images of the controls without and with irradiation of Cs-137, respectively. **C** and **D** the images of the GNPs-uptake cells without and with irradiation, respectively. Kit for mitochondria: MitoCapture^TM^ (yellow). Kit for nuclei: Hoechest 33342 (blue).


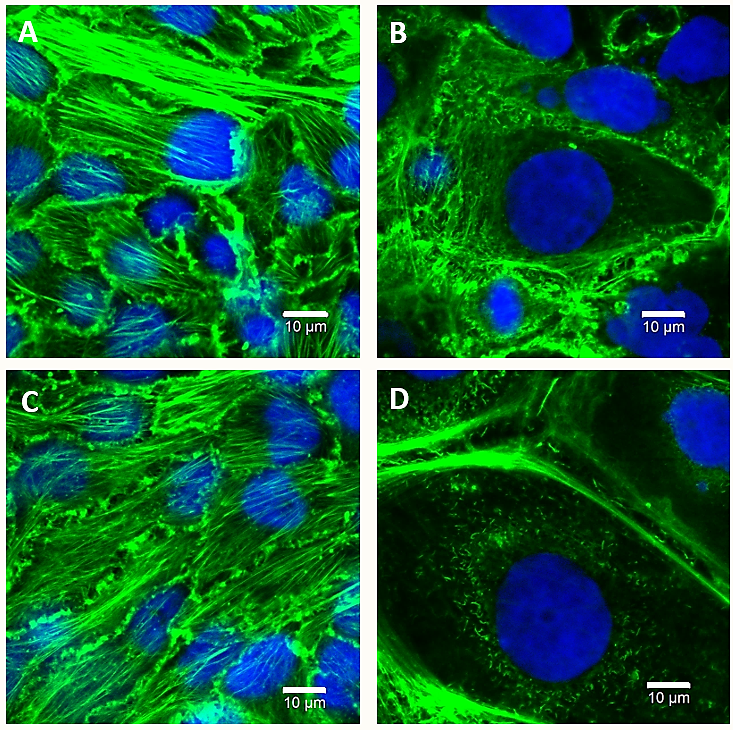


**Fig. S3** Cell fluorescence images of LSCM for labeled cytoskeletons, 48 hours after the irradiation of Cs-137 with a dose of 6 Gy (magnification: ×100). **A** and **B** are the images of the controls without and with irradiation of Cs-137, respectively. **C** and **D** the images of the GNPs-uptake cells without and with irradiation, respectively. Kit for cytoskeletons: Alexa Fluor^TM^ 488 Phalloidin (green). Kit for nuclei: Hoechest 33342 (blue).
